# Annotations

**Published:** 1899-11-04

**Authors:** 


### ANNOTATIONS.

### An Eel in a Water-pipe.

A sudden death recently took place near Charing Cross, and although the circumstances attending it were such as to suggest gross and culpable carelessness, we are afraid that no inquest will be held, for "the deceased" was but an eel. Yet the event deserves investigation. The story as it comes to us in the morning papers is that one day last week the Constitutional Club suffered from a sudden obstruction to its water supply, and that on the cause of this obstruction being investigated it was found to be due to the impaction of a good sized eel in the water main. The question is, How did it get there? Clearly no eel, however slippery it might be, could wriggle its way into a water main; and the presumption is that, although fat and plump when it became famous by stopping the water supply, and interfering with the "washing-up" at the Constitutional Club, it had been but a tiny creature when it escaped the meshes of the filter and thus gained access to the pipe. And this is where our whole interest in this eel centres. London is supplied with filtered water. So splendidly is it filtered that hardly a microbe can get through; and however vile the water of the river may be—as, indeed, it often is—we need not fear, for the filtration to which this vile stuff is subjected keeps back all that is harmful, and lets nothing but pure water pass. At least, so we are told; how, then, about this little eel? Not that we object particularly to the eel, but we do object very strongly to a filter that will let eels through; or, what is the same thing, to a filtering process so carelessly carried on that unfiltered water is sometimes allowed to pass into the pipes. It is as a means of keeping out microbes and such-like causes of disease, that filtration is made compulsory. But, by the side of such micro-organisms, even the smallest eel is a perfect monster; and, if an eel can pass the filter, whole regiments of disease germs can march through with the most perfect ease. It has been shown that distinct increase in the notifications of typhoid fever has followed the occurrence of floods above the intakes of the water companies, which ought not to be the case if the filtration were as perfect as it is said to be. But how can we wonder if eels can get through?

### Prussic Acid for the Criminal.

America invented the electric chair for the termination of the criminal's career, but America is not yet content. A new mode of execution is now suggested, one which is as nearly painless as possible, and which would deprive the criminal's death of even those mental horrors from which no form of execution hitherto employed has been free. The plan is that the condemned cell shall be made a sort of lethal chamber, into which hydrocyanic gas can be pumped by an apparatus outside. The gas would speedily stupify the condemned, and he would pass unconsciously into death without even knowing how it came. The criminal need not even know the hour at which he was to die. Such a death would be so merciful that we might ask Nature, which is by no means so considerate, to take example by it, and treat decent folks as kindly as the law would the criminal. The question arises, Would such a mode of execution have any restraining power on potential murderers? The main intention of all punishment is not only to take vengeance on the criminal, but to prevent others from copying his crime. In some cases we know that all the horrors of an Oriental torture-chamber would not suffice to restrain a murderer's hand, but there are others who are certainly kept from the worst of crimes by fear of the probable punishment. Is it wise, then, to make that punishment, if inflicted, too easy, too painless a mode of passing into that eternity in which most criminals do not believe? Nay, further, would it be wise to hold before those would-be suicides, those neurotics who long to shuffle off this mortal coil yet dread the process, the offer of a painless and unconscious death, if only they can screw up their courage to the sticking point, and can summon up nerve enough to plunge a bare bodkin, not into their own breast but their neighbour's?

### Winter Clothing.

With the coming of November everyone thinks of winter clothing, and as a clothing we all believe in wool. Undoubtedly. As the saying goes, "there is nothing to beat it." A man clothed in wool is far nearer to the condition of the natural monkey with whom he is said to have such affinities than when dressed in any other garb, even than when got up in furs or in the homely sheepskin. The trouble is that wool is difficult to wash, and very difficult indeed to sterilise without deterioration, whereas cotton and linen can be made not only to look clean, but to be really clean, by the simple process of putting it in a copper and boiling it. If one inquires how woollen underclothing is dealt with by the ordinary laundress one finds that it is washed in warm water, well lathered with soap, and rapidly dried, preferably upon some form of stretcher, the aim being not entirely cleanliness but the avoidance of the shrinking which is apt to take place if it be left long in the water, and of the "going hard," which occurs if the fabric be boiled. The process is definitely laborious, and one that the poor find too tedious and expensive to be indulged in more frequently than is absolutely necessary. Hence, no doubt, what a contemporary calls the "dreadful smell of humanity" which characterises an English crowd, for England, of all countries, is the one whose inhabitants most affect woollen clothing. We wear clothing mainly for the sake of warmth; and, in this climate, woollen clothing is the safest because it serves best to keep us warm under all sorts of conditions. Cotton may be made to be nearly as warm as wool, so long as it is dry. But once it gets wet there is a different tale to tell, and therein lies its deficiency. If anyone could so change the fibres of cotton that they would not become sodden and limp when wet; or if anyone could so modify wool that it could be washed as easily and with as little deterioration as can be done with cotton, not only would he make his fortune, but we might see some chance of getting rid of that "dreadful smell of humanity" which pervades all public assemblies in these isles. The fault lies in the clothes far more than in the man.

